# Oxidation of Marine Omega-3 Supplements and Human Health

**DOI:** 10.1155/2013/464921

**Published:** 2013-04-30

**Authors:** Benjamin B. Albert, David Cameron-Smith, Paul L. Hofman, Wayne S. Cutfield

**Affiliations:** ^1^Liggins Institute, University of Auckland, Private Bag 92019, Auckland 1142, New Zealand; ^2^Gravida: National Centre for Growth and Development, University of Auckland, Private Bag 92019, Auckland 1142, New Zealand

## Abstract

Marine omega-3 rich oils are used by more than a third of American adults for a wide range of purported benefits including prevention of cardiovascular disease. These oils are highly prone to oxidation to lipid peroxides and other secondary oxidation products. Oxidized oils may have altered biological activity making them ineffective or harmful, though there is also evidence that some beneficial effects of marine oils could be mediated through lipid peroxides. To date, human clinical trials have not reported the oxidative status of the trial oil. This makes it impossible to understand the importance of oxidation to efficacy or harm. However, animal studies show that oxidized lipid products can cause harm. Oxidation of trial oils may be responsible for the conflicting omega-3 trial literature, including the prevention of cardiovascular disease. The oxidative state of an oil can be simply determined by the peroxide value and anisidine value assays. We recommend that all clinical trials investigating omega-3 harms or benefits report the results of these assays; this will enable better understanding of the benefits and harms of omega-3 and the clinical importance of oxidized supplements.

## 1. Introduction

Marine omega-3 rich oils (marine oils) are the most popular supplements in the United States; after a rapid rise in popularity, they are now used by more than a third of American adults [1, 2]. Marine oils (derived from fish, krill, shellfish, calamari, or algae) differ from terrestrial plant sources of omega-3 fatty acids such as flaxseed as they contain the long chain polyunsaturated fatty acids (LC-PUFAs), eicosapentaenoic acid (EPA), and docosahexaenoic acid (DHA). They show promise particularly in the prevention of cardiovascular disease [3], the treatment of inflammatory disease [4], improving early life neurodevelopment, preventing cognitive decline [5], and potential benefits to metabolism [6]. However, it is important to understand that unlike most pharmacological and neutraceutical interventions, these oils are highly susceptible to oxidation. There has been concern about the safety of oxidized fish oil since the 1950s [7], and although there is evidence that over-the-counter supplements are frequently oxidized, this has had no impact on the requirements for storage and labelling or on the design of human clinical trials. No human efficacy trials have reported the oxidative state of the trial oil which would question the validity of the results and conclusions of these trials. It is currently unclear to what degree the oxidation of fish oil influences its efficacy or harm in humans. This commentary discusses these issues and outlines the implications for interpreting the literature and improving clinical trial design.

## 2. How Stable Are Marine Omega-3 Supplements?

n-3 LC-PUFAs are chemically unstable, so that marine oils rapidly oxidize during storage to a complex chemical soup of lipid peroxides, secondary oxidation products, and diminishing concentrations of unoxidized fatty acids. As a result, the composition of a fish oil supplement cannot be simply inferred from the labelled EPA and DHA concentrations.

n-3 LC-PUFAs are highly prone to oxidation due to their large number of double bonds and their position within the fatty acid chain [8, 9]. This makes them prone to oxidation because bisallylic carbons, those between two double-bonded carbon atoms, have a low activation energy for hydrogen loss and free radical formation [8]. n-3 LC-PUFAs have more of these vulnerable bisallylic carbons (EPA : 4, DHA : 5) than the short n-3 PUFA (*α*-linolenic acid : 2) or n-6 PUFAS (arachidonic acid : 3) while the monounsaturated fatty acids and saturated fatty acids have none. In the presence of various initiators, a lipid radical is formed starting an expansive chain reaction which creates lipid peroxides and more radicals from unoxidized PUFAs. A complex array of different peroxide molecules arises depending on the position of the oxidized carbon, and after undergoing cis-trans isomerisation and a shift of double bonds, conjugated dienes and trienes are produced which have different polarity and shape to the original fatty acid [8]. A potentially important class of n-3 peroxidation products, the prostaglandin-like F3-isoprostanes and F4-neuroprostanes are formed from EPA and DHA, respectively [10]. Thus, the primary oxidation products of n-3 LC-PUFAs are chemically different from unoxidized n-3 LC-PUFAs and may have different biological properties. 

Lipid peroxides are unstable and further degrade to form secondary oxidation products including aldehydes such as 4-hydroxyhexenal (HHE) and malondialdehyde (MDA) [11]. As the oil oxidizes over time, there is an initial exponential increase in the concentration of lipid peroxides. These later degrade and the concentration of potentially harmful secondary oxidation products increases as the lipid peroxides decrease. 

The rate of lipid peroxidation is influenced by light, heat, and oxygen concentration even at normal room conditions. Moreover, even oil stored in the dark at 4°C may oxidize unacceptably within a month of storage [12]. Added antioxidants reduce but do not prevent oxidation [13]. The tendency of n-3 LC-PUFAs to oxidize under light is also influenced by the presence of impurities such as protein or heavy metals and its conjugate; phospholipids are more prone to oxidation than triglycerides [8]. Because peroxidation is an accelerating chain reaction, small concentrations of peroxides in the source oil, or exposure to oxidising conditions during processing could have a large effect on the rate of oxidation. In addition, deodourisation to remove fishy odour often involves high temperature which may accelerate secondary oxidation. Significant peroxidation is highly likely to occur in over-the-counter supplements which are commonly kept at room temperature both in retail shops and in the home.

Oil in an omega-3 supplement may differ substantially from the oil in fresh fish depending on its age, heat and light exposure. As a result, these supplements should be viewed as a complex mix of EPA, DHA, other fatty acids, additives, and an unspecified concentration of potentially toxic lipid peroxides and secondary oxidation products. 

## 3. Can Oxidation Be Easily Quantified and Reported?

Measurement of specific lipid peroxide species and secondary oxidation products requires gas-chromatography mass-spectrometry [14–17] or other chromatographic techniques [9] which are expensive and require significant technical expertise. However, the oxidative status of supplemental oils can be easily estimated using the peroxide value (PV) and anisidine value (AV) assays. While these are nonspecific, they are repeatable, simple, and cost effective, and guidelines exist for recommended maximum levels in marine omega-3 supplements. The peroxide value (PV) is a simple titration enabling quantification of the concentration of peroxide groups in oil [18] while the anisidine value (AV) is a colorimetric test which enables estimation of the concentration of secondary oxidation products. Both measurements are required to estimate total oxidation (TOTOX = 2 × PV + AV). 

A number of organisations have endorsed maximum recommended levels of oxidation in supplements [19–22]; though due to the paucity of human evidence, these are based on palatability and not the effect on human health [20]. Clearly, this implies a need for evidence-based guidelines. The simplest way to improve the knowledge base would be for clinical trials to report the oxidative status of their trial oils, so that benefits and harms could be associated with the oxidative state. 

## 4. Are Over-the-Counter Marine Omega-3 Supplements Significantly Oxidized?

The oxidative states of retail oils are not routinely labelled and it is surprising that there has not been more formal evaluation of the oxidative stability of marketed omega-3 supplements. When over-the-counter supplements have been investigated, the frequency of excess oxidation [19–22] was highly variable but not uncommon, affecting between 11%–62% of products [23–27]. Thus, consuming purchased supplements entails risk of exposure to unacceptably oxidized oil, and it is likely that the omega-3 supplements used in many clinical trials have also been significantly oxidized. Understanding the effects of oxidized omega-3 LC-PUFAs on health is thus important both for the vast number of supplement consumers and for scientists and clinicians interpreting the medical literature.

## 5. Are Oxidized Omega-3 Oils Efficacious?

To our knowledge, no clinical trial investigating the efficacy of omega-3 in humans has reported the oxidative state of the trial oil or compared oxidized and nonoxidized oils. The relative efficacy of highly oxidized and nonoxidized oil cannot be inferred. However, it is likely that there is a difference.

The mechanisms of action of omega-3 are not fully understood, but there are multiple interacting mechanisms including acting as a ligand for intracellular and extracellular receptors, competition for metabolism by enzymes, structural roles in cell membrane, and stearic interference with ion channels. For illustration, triglyceride lowering is mediated by interaction with sterol receptor binding protein 1-c (SREBP1-c) and the peroxisome proliferator activated receptor alpha (PPAR-*α*) [28]. Anti-inflammatory, hypotensive, and antiplatelet effects may be mediated by competition with arachidonic acid for synthesis of eicosanoids by the enzyme cyclooxygenase [3]. Antiarrhythmic effects are in part due to stearic interference with ion channels [29]. Insulin sensitisation is partly mediated by interaction with PPAR-*γ* an intracellular transcription factor [6] and binding to the recently discovered G-protein linked receptor GPR120 on the cell surface [30]. Omega-3 fatty acids may also have anti-oxidant effects [31] and influence cell membrane fluidity [32].

As lipid peroxides have different shape, polarity, and reactivity to their parent fatty acid, it is likely that they will be ineffective through some if not all of these mechanisms. Because these mechanisms are diverse, the effect of oxidized supplements may be divergent, some beneficial effects may be lost but not others; lipid peroxides may even have their own unique functions.

Surprisingly, there are no specific clinical trials investigating the effects of oxidation on the efficacy of marine n-3. However, in a clinical trial of fish oil supplementation with and without the anti-oxidant vitamin E, triglycerides decreased significantly more in the vitamin E group [33]. Increased efficacy with vitamin E is most likely due to prevention of oxidation of the oil either prior to consumption or *in vivo*. Interestingly, in a study of liver tissue in culture, oxidized EPA inhibited the inflammatory NF-*κ*B pathway [34]. This may be mediated by n-3 derived isoprostanes, as these peroxides have been shown to be biologically active, inhibiting macrophage NF-*κ*B activation in tissue culture [35]; and affecting vascular and platelet function [10]. It is not yet clear whether these effects are important *in vivo*; however, they provide evidence for a divergence of effects when n-3 LC-PUFAs are oxidized. Clearly, the effect of oxidation on efficacy of omega-3 requires more investigation; at minimum, the oxidative state of supplements used in clinical trials must be reported. Further, detailed studies are also required to establish both the bioavailability of individual oxidized lipid species and to provide greater insights into their biological functioning.

## 6. Are Oxidized Omega-3 Supplements Harmful?

There are insufficient interventional human studies that examine potential biological functions of oxidized marine n-3; however, there is evidence that lipid peroxidation is involved in human disease. In addition, animal studies show that oxidized lipids may cause organ damage, inflammation, carcinogenesis, and advanced atherosclerosis. These deleterious effects cannot be ignored, particularly when marine n-3 is taken during vulnerable stages of life such as pregnancy, early childhood, and old age and for long periods of time.

Lipid peroxides are absorbed through the gut and incorporated into chylomicrons [36], LDL [37], and VLDL [38]. Their active transport in LDL particles and particularly subsequent oxidation of LDL may be important in atherogenesis [11, 39]. Lipid peroxides also partially decompose to secondary oxidation products in the gut which are absorbed [40].

Lipid peroxides hasten oxidation of other fatty acids to create further lipid peroxides in an expansive chain reaction. We speculate that ingested omega-3 peroxides could lead to lipid membrane peroxidation, cell damage, and oxidative stress, which are known to be mechanisms of disease. Endogenous membrane lipid peroxidation results in altered membrane fluidity, transport, and cell signalling [8] which also may be an important disease mechanism. For example, acute severe lipid and protein peroxidation has been shown to be the cause of death when, despite appropriate treatment, people die from organophosphate poisoning [41]. Chronic lipid peroxidation may be a mechanism in carcinogenesis [42] and in the pathogenesis of Alzheimer's disease where the secondary oxidation product 4-hydroxynonenal (HNE) appears to have a role in both the formation of neurofibrillary tangles and neurotoxicity [43]. Oxidative stress further activates the NF-*κ*B pathway and increases production of proinflammatory cytokines [44]. Chronic low grade inflammation is involved in degenerative disease including atherogenesis [45] and the generation of insulin resistance in the metabolic syndrome [46].

Animal studies provide clear evidence that oxidized lipids are harmful, though typically using higher doses of oil than humans consume or administering oxidation products in nonphysiological ways [11]. Chronic feeding of oxidized PUFAs to rats led to growth retardation, intestinal irritation, liver and kidney enlargement, haemolytic anaemia, decreased vitamin E, increased lipid peroxides and inflammatory changes in the liver, cardiomyopathy, and potentially malignant colon cell proliferation [11]. A major secondary oxidation product of omega-3 oils is the aldehyde HHE. HHE when injected into the peritoneum causes necrotising peritonitis and when injected intravenously causes liver damage. It is chemically similar to the better studied omega-6 oxidation product HNE which is known to be highly toxic and causes DNA damage [11, 42]. 

There is increasing evidence that *in vivo* oxidation of LDL has a role in atherogenesis [47]. Unmodified LDL cannot induce foam cell formation; however, after oxidative modification it can be recognised by the scavenger receptor of macrophages and is rapidly absorbed [9, 48]. Given that ingested peroxides are transported in LDL [37], it is possible that they could have a role in enhancing LDL oxidation and atherogenesis. This is supported by a study in rabbits where addition of fish oil to a high cholesterol diet led to rapid atherosclerosis [49]. We speculate that if this is due to oxidation of LDL, ingested oxidized marine n-3 could be atherogenic in humans. This could contribute to the disappointing results in primary and secondary cardiovascular prevention trials [50] and requires further investigation.

Consuming marine oil leads to increased plasma [33] and urinary [51] MDA in humans and mice, due to both absorption of peroxidized oil and *in vivo* oxidation with subsequent degradation of peroxides [51]. This is only partially reduced by addition of antioxidants [51–53]. MDA induces transition, transversion, and frame shift DNA mutations [54]. It has been shown to cause thyroid tumours when fed to rats and skin cancer with topical application [55]. The little evidence in humans is unclear; however, women with breast cancer have higher concentrations of MDA-DNA adducts in their normal breast tissue than controls, consistent with MDA exposure increasing risk [56]. 

One human-randomized placebo-controlled trial has examined the effects of oxidized versus nonoxidized oil over 7 weeks [57]. No difference was found in markers of *in vivo* lipid peroxidation (urinary 8-isoFGF2*α*, plasma HHE and HNE), markers of antioxidant activity, C-reactive protein, or liver function tests. This suggests that oxidized marine n-3 may not be associated with acute oxidative toxicity. However, this is not reassuring as the study was short and did not assess important pathological markers associated with atherosclerosis such as oxidized LDL or carotid artery intimal thickness. Further, there was no assessment of specific inflammatory markers such as prostaglandins and cytokines or of markers of DNA damage. Thus, the risks of atherosclerosis, DNA damage, malignancy, and inflammation especially at tissue level remain open. If low grade, chronic peroxide, aldehyde, or MDA exposure is important in disease it may require long periods of followup to identify an effect. Some pathological effects such as tissue level inflammation may be difficult to detect without invasive methods such as muscle, liver, or adipose tissue biopsy. 

In summary, given the paucity of specific evidence, it is currently impossible to know whether marine oils, some of the world's most popular supplements, are safe after oxidation. The effects of oxidation on the biological effects of these oils may be complex, there could be both beneficial [10, 34, 35] and harmful effects. Thus, long-term safety studies of marine oil are required, looking at appropriate disease outcomes and surrogates and relating these to the oxidative state.

## 7. Why Is the Omega-3 Supplementation Literature Conflicting?

The omega-3 supplementation literature is highly conflicting, especially in the area most heavily researched, the effect on cardiovascular disease. Oxidation may be a major cause of these conflicting results; however, it has never been reported in these trials.

Epidemiological studies link higher dietary [58–64] or plasma n-3 LC-PUFAs [63, 65–67] to lower risk of diabetes and cardiovascular disease. Furthermore, supplementation with encapsulated fish oil or fortified foods improves a wide range of cardiovascular risk factors including lipid profile [68–73], blood pressure [69, 74, 75], heart rate [76] and variability [77, 78], platelet aggregation [79, 80], endothelial function [81], and atherosclerotic plaque stability [82]. After myocardial infarction, fish oil reduces sudden cardiovascular death probably due to an antiarrhythmic effect [29, 83–85]. Systematic reviews of minor outcomes such as blood pressure [86, 87] and plasma triglycerides [88] are overall positive; however, individual studies are mixed. Moreover, despite the abundant evidence for improvement of cardiovascular risk factors, the results of primary and secondary prevention trials have been conflicting [89–91], and a recent systematic review found no overall effect of marine oil supplementation on the risk of all-cause mortality, cardiac death, sudden death, myocardial infarction, or stroke [92]. In explaining the conflicting effects of marine oil on health, authors have overlooked oxidized supplement as an explanation. Alternative explanations include a true lack of efficacy, obscuration of benefit by other cotreatments that improve cardiovascular risk such as statins, aspirin and beta-blockers [50], high background fish intake in some populations [93], and underpowered studies. However, it must be recognised that the oxidative status of the trial oil could also explain these disappointing results. If oxidized oils are less efficacious, or if they cause harm, for example, by advancing atherosclerosis then provided some studies used oxidized supplements, these results would be expected. We are currently in danger of concluding that marine n-3 supplements are ineffective in the prevention of cardiovascular disease, before they have been adequately investigated. 

## 8. What Are the Implications for Interpretation of the Literature and Future Clinical Trials?

To assess the degree to which the importance of oxidation of marine oil is understood, we identified all human clinical trials published in 2012 using Pubmed. Of 107 reports, only one study investigating short-term harm reported the oxidative state of the trial oil (previously described) [57]. This strongly suggests that the instability of marine oil is generally unrecognized or not considered important. 

It is currently impossible to determine how oxidation affects the efficacy or potential harms of marine oil. This makes interpretation of the clinical trial literature problematic. If the oxidative state of marine oils may affect efficacy or harm, then physicians should recommend, and consumers select, a supplement with the same oxidative state as the oils used in clinical trials that have shown benefit and safety. This is currently impossible because although over-the-counter-supplements are frequently oxidized [23–27]; the oxidative state of trial oils and retail supplements remain unreported. 

That marine oils have beneficial effects on many indices such as plasma triglycerides, blood pressure, inflammation, and insulin sensitivity (in rodents) is not in question. The purpose of this commentary is to highlight the limited knowledge about the importance of oxidation to these effects. For example, some *in vitro* and animal studies have stored oil under conditions likely to prevent oxidation such as under nitrogen or at very low temperature [94–97]. This confirms for example, that unoxidized marine oil prevents insulin resistance in the rat [95]. However, whether oxidized oil has the same effect is unknown. In contrast, emerging evidence has shown that some *in vitro* anti-inflammatory effects are solely mediated by oxidized oil, but the clinical relevance of this is unclear.

Future safety and efficacy trials, particularly in humans, should report the oxidative state of the marine oil. This could most easily be done by reporting the peroxide, anisidine, and TOTOX values. Even established benefits of marine oil need to be reinvestigated with provision of this information. In parallel, there should be a move to labelling marine oil supplements with these same oxidative indices and a production and storage chain that minimizes oxidation prior to purchase. Only then can we generalise efficacy and safety trial data to the available omega-3 supplements and provide informed recommendations to patients and consumers.
